# Physical activity has a stronger correlation with arterial stiffness than strength, balance, or BMI in an older population

**DOI:** 10.3389/fragi.2023.1279479

**Published:** 2023-12-14

**Authors:** Hannah Hill, Catherine A. Elliot, Catherine A. Lizamore, Michael J. Hamlin

**Affiliations:** ^1^ Department of Tourism, Sport, and Society, Lincoln University, Lincoln, New Zealand; ^2^ Department of Applied Science and Social Practice, Ara Institute of Canterbury, Christchurch, New Zealand

**Keywords:** arterial stiffness, older adult, exercise, pulse wave velocity, balance, strength, fitness, hypertension

## Abstract

**Background:** Arterial stiffness is associated with an array of debilitating health conditions. While exercise typically has beneficial effects on both arterial stiffness and overall health, more research is needed to understand the associations of different types of fitness indices with arterial stiffness.

**Aim:** To investigate the relationship between balance, strength, cardiovascular fitness and physical activity with arterial stiffness (as measured by pulse wave velocity (PWV)) in older adults.

**Method:** Eighty retirement-village residents (24 males, 56 females, age: 78.2 ± 6.4 years, weight: 69.4 ± 12.5 kg, height: 162.9 ± 8.5 cm) completed the Yale Physical Activity Survey, PWV measurement, 30-s sit-to-stand leg strength test, hand grip strength assessment, 4-stage balance test, and a 6-min walk fitness test. The number of exiting risk factors (smoking, previous heart incidents, previous stroke(s), having hypertension, or taking anti-hypertension medication) were tallied. Pearson’s correlations were used to assess the relationship between PWV and health and fitness parameters. Results were interpreted using qualitative inference.

**Results:** The number of risk factors (r = 0.57, *p* < 0.001), age (r = 0.51, *p* < 0.001) and systolic blood pressure (r = 0.50, *p* = 0.001) had strong, harmful associations with PWV. Total physical activity minutes/week (r = −0.31 *p* = 0.01), total energy expenditure Kcal/week (r = −0.30, *p* = 0.01), and the 6-min walk test (r = −0.29, *p* = 0.01) had a moderate, beneficial association with PWV, while sit-to-stand (r = −0.27, *p* = 0.02) and balance (r = −0.27, *p* = 0.01) had a weak, beneficial association with PWV. Hand grip strength (r = 0.02, *p* = 0.94) and body mass index (r = −0.04, *p* = 0.75) had no significant associations with PWV.

**Discussion:** All measured fitness indices had beneficial associations with PWV. However, having more risk factors, increased age, and higher systolic blood pressure had significant (harmful) associations with PWV in our older population.

**Conclusion:** Controlling cardiovascular risk factors, especially high systolic blood pressure, is likely to have the largest beneficial effect on PWV. Improving general physical activity, including walking capacity, may prove beneficial in improving PWV in an older population.

## 1 Introduction

In New Zealand, as in many developed countries, the older adult population is the fastest growing age group, Statistics New Zealand, 2019, with many of these individuals making the shift to live in community dwelling retirement villages. While living in residential care (in rest home or respite care) is associated with higher sedentariness (Ministry of Health, 2013), there is a lack of research on the association between physical activity and health outcomes in individuals living independently in community-style retirement villages. Increasing our understanding of the health correlates of this population will go towards improving wellness and extending disability free life years in this population.

Given the damaging effects that cardiovascular decline can have on quality of life and independent living in older adults, a considerable amount of research has been conducted to determine the best means of delaying or reducing arterial stiffness. Chronological aging has been identified as one of the main clinical determinants of central artery stiffness and it is especially pronounced among adults aged 50+ (Tsai et al., 2017; Ceciliato et al., 2020) for both men and women (van der Heijden-Spek et al., 2000). Moreover, the PWV increase in people with hypertension has been found to be higher (0.93 m/s per decade, *p* < .001) than in normotensive people (0.44 m/s per decade) (Diaz et al., 2017).

Although it is impossible to stop the natural process of aging, there are factors that may help to slow this age-associated increase in arterial stiffness. A reduction in sedentary behaviour has been found by Ahmadi-Abhari et al. (2017) to be highly associated with a slowing of the natural progression of age-related stiffening of the arteries. In older populations prone to increased arterial stiffness (Wen et al., 2015), regular physical activity is an essential component of healthy aging (Chodzko-Zajko et al., 2009). To best support healthy aging, national health organizations, such as New Zealand’s Ministry of Health, recommend the inclusion of aerobic**,** resistance, and balance components to exercise routines targeted at older populations (Chodzko-Zajko et al., 2009; Ministry of Health, 2013). These recommendations are typically supported by current research, for example, habitual physical activity from mid-life to late-life is associated with lower central arterial stiffness and pulse pressure in older adults (Tanaka and Safar, 2005). Similarly, healthy active people with high maximal oxygen uptake, and few cardiovascular disease risk factors have considerably lower central pulse wave velocity than individuals who are sedentary with cardiovascular disease risk factors (7.0 ± 1.1 and 8.2 ± 1.4 m/s mean ± SD respectively) (Dobrin, 1983; Deiseroth et al., 2019).

The heterogeneity in aging and physical ability (from regularly and independently physically active to frail and inactive) makes it difficult to prioritize the exact types of physical activity, or combinations of physical activity components needed for optimal health benefits (Bangsbo et al., 2019). Regardless, some studies have attempted to tease out more guidance regarding different exercise modalities effects on arterial stiffness which would be important for the older population.

Regular aerobic exercise improves arterial stiffness significantly, with larger affects among those starting with stiffer arteries (PWV ≥8 m/s). However, resistance exercise had no effect on PWV nor did the combined aerobic plus resistance exercise (Ashor et al., 2014). While research has typically found resistance training to be ineffective in reducing arterial stiffness (Ceciliato et al., 2020), some have found that while high-intensity resistance training is unlikely to improve arterial stiffness, low-intensity resistance training or whole-body vibration training may be helpful in reducing carotid–femoral PWV (Jurik et al., 2021).

For older adults, balance is important and when balance is compromised their ability to live independently reduces. While studies investigating the effect of balance, or balance exercises are uncommon, there is some evidence to suggest that improving both core strength and balance is more effective in reducing arterial stiffness than resistance training (Kujawski et al., 2018), perhaps due to the strong association between physical activity levels, balance and core strength. Furthermore, older adults (over 65 years old) with higher PWV (>10 m/s) appear to have more difficulty in maintaining their balance when compared to those with lower PWV, even after controlling for age (Peultier-Celli et al., 2021). Again, it could be that better balance comes as a result of, at least in part, its association with higher physical activity levels.

Which health variables are most important for arterial health of older adults continues to be debated. Therefore, the aim of this study was to investigate the relationship between strength, balance, cardiovascular fitness, and physical activity levels to determine which health variables can predict arterial stiffness in an older population residing in community dwelling retirement villages.

## 2 Materials and methods

### 2.1 Participants

Residents in independent-living retirement homes were invited to participate in the present study through recruitment posters, notice boards and flyers, as well as through information presentations and word-of-mouth communication between residents. Inclusion criteria encompassed: being over the age of 65 years, living independently in a retirement village in Canterbury, New Zealand, and being physically able to participate in all fitness tests (after approval by the participants’ general practitioner). Although 28 participants (35% of sample) had cardiovascular disease (CVD), all participants, including those with heart incidents (including heart failure and chronic artery disease (CAD) and previous stroke were able to perform the laboratory tests. Exclusion criteria included inability to attend the appointment, or on the advice of the individual’s medical professional. Following an expression of interest, each prospective participant was provided a research information sheet, as well as the written informed consent document and physical activity survey. Participant recruitment was finalized by arranging a time for testing with the researcher via phone or email. Ethical approval was completed by the local university’s Human Ethics Committee (reference number 2017–34), and all participants provided written informed consent prior to their participation.

Ninety-five participants living in independent residential units from 3 different retirement villages were recruited into this study. Of the 95 participants initially recruited, 15 participants were excluded due to incomplete datasets (missing all anthropometric and physical activity testing data: n = 2; missing sit-to-stand data, n = 1; missing sit-to-stand and PWV data, n = 1; missing PWV data: n = 9; incomplete 6-min walk test, n = 1; incomplete survey data, n = 1). Therefore, the final dataset comprised 80 participants from 3 different retirement villages (Village A: n = 44; Village B: n = 5; Village C: n = 31; see Table 1 for participant information; Table 2 for differences in selected variables in participants from different villages).

**TABLE 1:** Means and standard deviations of participant demographics, and frequencies of risk factors in participants.

	All participants (n = 80)
Sex	Male: 24; Female: 56
Age (years at time of measurment)	78.2 ± 6.4
Systolic Blood Pressure (mmHg)	133.6 ± 15.0
Diastolic Blood Pressure (mmHg)	74.9 ± 9.7
Weight (kg)	69.4 ± 12.5
Height (cm)	162.9 ± 8.5
Body Mass Index	26.1 ± 4.1
**Risk Factors**	
Smoker	n = 2
Heart incidents	n = 22
Previous stroke	n = 6
Systolic blood pressure ≥140 mmHg	n = 29
Blood pressure medication	n = 45

**TABLE 2:** Differences in age, pulse wave velocity, systolic blood pressure, body mass index, and number of risk factors between the residents of different villages.

Retirement Village^a^	Age	Pulse wave velocity^b^	Systolic blood pressure	Body mass index	Number of risk factors^c^
	(Years, *M* ± *SD*)	(ms, *M* ± *SD*)	(mmHg, *M* ± *SD*)	(*M* ± *SD*)	(*M* ± *SD*)
A (n = 44)	76.3 ± 6.1	11.1 ± 2.4	130.2 ± 13.0	25.5 ± 4.1	1.1 ± 1.1
B (n = 5)	84.4 ± 2.7	12.6 ± 2.8	139.6 ± 18.8	24.2 ± 4.0	1.6 ± 1.1
C (n = 31)	79.8 ± 6.3	12.8 ± 2.13	137.5 ± 16.3	27.3 ± 3.9	1.5 ± 1.1
ANOVA	*F* (2,77) = 5.87, *p* = 0.00^d^	*F* (2,77) = 4.87, *p* = 0.01^d^	*F* (2,77) = 2.74, *p* = 0.07	*F* (2,77) = 2.57, *p* = 0.08	*F* (2,77) = 1.90, *p* = 0.16

^a^ Participants were recruited from 3 local retirement villages.

^b^ Carotid- Femoral pulse wave velocity was measured to assess arterial stiffness.

^c^ Number of risk factors included: Smoking, any previous heart incidents, previous stroke, systolic blood pressure ≥140 mmHg, and whether they were on blood pressure medication.

^d^ Statistically significant.

### 2.2 Procedures

All testing was conducted at a location within the participant’s village, in a large, quiet, temperature-controlled room made available by the village administrators. Each testing appointment lasted approximately 1 h and included an oral review of the Physical Activity Readiness Questionnaire (PAR-Q), a general health questionnaire, the YALE physical activity Survey, an arterial stiffness measurement, a strength test, a balance task, and a cardiovascular fitness test. All the tests were described verbally, and then demonstrated to the participant prior to their completion by the participant, however, due to time constraints, there was no familiarization session offered.

#### 2.2.1 Yale physical activity survey

The Yale Physical Activity survey was used to gather information on the physical activity of the older participants in this study. The Yale Physical Activity survey has been specifically designed for an older population and includes many of the lower intensity lifestyle activities that are often omitted by surveys designed for a younger population (Kruskall et al., 2004). The survey is composed of two sections. The first section examines typical physical activity patterns (work, exercise, and recreational) over a typical week in the previous month. Each of these activities was then quantified into minutes of activity and is multiplied by an intensity code to create an energy expenditure estimation. All energy expenditure estimations were added together to create an overall physical activity energy expenditure estimation (kcal/wk). In the second part of the survey, participants examined their physical activities over the previous month, and the activity was divided into vigorous activity, leisurely walking, moving, standing, and sitting. The Yale Physical Activity survey has moderate test-retest reliability (Gennuso et al., 2015), and has reasonable validity against objective measures of energy expenditure from physical activity (Kruskall et al., 2004). For the purposes of this analysis, total time spent in physical activity in a typical week (as calculated in Kcal and MET min/week) as identified in the survey was used as a measure of physical activity volume.

#### 2.2.2 Arterial stiffness—Central pulse wave velocity

Arterial stiffness was measured using a SphygmoCor^®^ XCEL (AtCor Medical Pty Ltd, 2016), which has been validated against the well-validated tonometry-based SphygmoCor^®^ XCEL device (Hwang et al., 2014). Participants were asked to abstain from tobacco and alcohol for 6 and 12 h prior to the assessment, respectively according to the SphygmoCor^®^ XCEL Operators Manual (AtCor Medical Pty Ltd, 2016). Participants were asked to continue taking their medications as prescribed on the days leading up to testing as well as the morning of the testing. The participants were also urged to avoid eating for 6 h prior to the assessment, however, where this was not possible (i.e., food required with medication), a light meal was permitted as suggested in the SphygmoCor^®^ XCEL Operator’s Manual V1 (AtCor Medical Pty Ltd, 2016).

During the session, the participant’s height and weight were measured and entered into the SphygmoCor^®^ XCEL Software after which they were directed to a bed (no pillow) and asked to lie down in a supine position. Participants rested for 5 min during which correctly-sized brachial and femoral blood pressure cuffs were fitted. The brachial cuff was placed in line with the participant’s brachial artery, and the femoral cuff was positioned as high up on the participant’s leg as possible near the femoral artery. The direct method of measurement was used and is explained on page 47 of the SphygmoCor^®^ XCEL Operator’s Manual (AtCor Medical Pty Ltd, 2016). After both cuffs were fitted correctly, the researcher palpated the area of strongest carotid pulse, and marked the site with a highlighter. The participant was then asked to lift the knee of the leg with the femoral cuff to determine a more precise location of the femoral artery at the mid-inguinal point (MIP). Then, the leg was laid flat and a tape was used to measure the actual distance between the MIP and the top of the femoral cuff (∼90–180 mm). This more accurate measurement was used entered into the “femoral to cuff” area instead of the default 200 mm distance. The Sphymocor^®^ software then automatically subtracted the MIP-femoral cuff measurement from the total distance measured using an infant stadiometer (SECA 707, Hamburg, Germany) from the carotid artery point to the top of the femoral cuff (carotid to cuff).

For the Pulse Wave Analysis measurement, the brachial cuff was inflated and automatically assessed systolic and diastolic blood pressure. This was then repeated to help account for any initial white coat syndrome. The second measure taken was entered into the software as it is required for the PWV to be measured. PWV was measured using a tonometer (SphygmoCor^®^ XCEL device) placed on the same side of the neck as the thigh cuff at the highlighted site of the strongest carotid pulse. Once the detection of a clear and consistent carotid waveform was achieved, the femoral cuff was inflated automatically, and femoral and carotid waveforms were analysed simultaneously by the SphygmoCor^®^ XCELSystem. Waveform quality was automatically assessed by the SphygmoCor^®^ XCEL software based on consistent pulse peaks, troughs and amplitude, and suitable data were indicated with a “quality-controlled” tick by the SphygmoCor^®^ XCEL software during the 10-s “capture” period. Only one measurement was taken unless the quality-controlled tick was not achieved, whereby the measurement was repeated until the tick was achieved. Since most participants had large neck physiology and stiffer skin (prone to sagging), it was not conducive to a consistent and stable PWV measurement, thus a quality control tick was very difficult to achieve over the 10 s period and up to ten PWV measurements were attempted, lasting up to 50 min total. In light of this surprisingly regular occurrence among our participants, in several instances, second PWV measures were attempted, but not achieved.

#### 2.2.3 Strength tests

##### 2.2.3.1 30-S sit-to-stand test

The 30-s sit to stand test (Rickli and Jones, 1999) provides a reasonably reliable and valid indicator of lower body strength in older adults (Rickli and Jones, 1999). To complete this test, the participant was seated in a chair and asked to fold their arms across their chest. The participant was asked to rise into a standing position and lower themselves back down into the chair again (one repetition). Participants were asked to complete this sit to stand movement as many times as possible over a single period of 30 s. The total repetitions comprised the participant’s leg strength score (Stevens and Phelan, 2013).

##### 2.2.3.2 Hand grip strength

A mechanical hand-grip dynamometer (Evernew, Japan) was placed in the participant’s dominant hand, with their elbow bent to 90°. Each participant was advised to avoid breath-holding before squeezing the handle with maximum effort. The best of three attempts was recorded. The procedure was repeated in the non-dominant hand. The participant’s highest score from each hand was combined and used as their hand-strength score.

#### 2.2.4 Balance test

To assess balance, the 4-stage balance assessment designed by the National Centre for Injury Prevention and Control was used (Stevens and Phelan, 2013). During this assessment, the participant progressed through a series of 4 poses of increasing difficulty with shoes on (no heels). Poses included: 1. Standing with feet side-by-side, 2. Standing in semi-tandem stance (placing the big toe of one foot against the instep of the other), 3. Standing with the toes of one foot touching the heel of the other, and finally, 4. Balancing on one foot (of their choosing) (Stevens and Phelan, 2013). Participants were asked to keep their eyes open during the tests. No participants required a cane or walker. Participants were required to successfully hold a pose for 10 s before progressing to the next pose. The test was stopped if a person needed to step out of the pose within the 10-s period. The highest level achieved was noted as the participant’s score.

#### 2.2.5 Cardiovascular fitness test: 6-min walk test

Participants completed the 6-min walk test along a 30-m long quiet corridor, where the participants were asked to cover as much ground as possible within the 6 min. The guidelines for the 6-min walk test are outlined in the statement by the American Thoracic Society (2002). To avoid biasing the outcome of the test, the researcher restricted communication with the participant to mark each passing minute, and to advise when there were 30 s of the test remaining. The total length (to the nearest m) completed by the participant in 6 min was recorded.

#### 2.2.6 Statistical analysis

To ascertain any potential variance in participants between the three villages (as there are physical fitness classes offered at some villages and not others), a single factor ANVOA was conducted on PWV, age (years, at time of data collection), number of risk factors (listed in Table 1), systolic blood pressure (SBP) and body mass index (BMI) (weight (kg)/height (m^2^)) in SPSS Version 26 (IBM Corp, 2019). This was a screening to indicate if there was a “village effect” that would need to be considered as a covariate in the main analysis. Pearson’s correlations were used to examine the relationship between PWV and selected physical ability measures (balance, 6-min walk test, sit-to-stand test, and grip strength) as well as age, BMI and SBP. Results were interpreted quantitatively using *p*-values, as well as qualitatively, using spreadsheets to estimate their magnitude of effect (Hopkins, 2007).

Qualitative interpretation ranged from the highest meaningful impact (strongly compatible) to the weakest impact (weakly compatible). Where no definitive qualitative interpretation was possible, an “unclear” result and recommendation for more data was made.

Finally, a multivariate regression was conducted on three groupings of variables to describe associations (correlations) and to model relationships (regressions) that each set of dependent variables and covariates have with PWV. In Model A, all the physical activity testing variables (sit-to-stand, balance, 6-min walk, and hand grip strength) were entered and analysed to determine their ability to predict PWV. In Model B, the regression was run on all variables measured in model A, but also added the reported covariates (BMI, number of health risk factors, SBP, age, sit-to-stand, balance, 6-min walk, hand grip strength, total min/week, and total Kcal/week to determine their combined ability to predict PWV. Finally, in Model C the significant predictors from the previous regression results (age, SBP, and number of risk factors) were analysed in a multivariate regression analysis to determine their ability and strength to predict PWV best when less influential variables were excluded.

## 3 Results

There was a wide degree of variability between the participants in each of the villages, particularly regarding their age and PWV (Table 2). Pearson’s correlations were run for each of the health variables individually with PWV (Table 3).

**TABLE 3:** Pearson’s correlations between pulse wave velocity and health variables.

	r-value	*p*-value	Qualitative inference	Effect on PWV
Number of risk factors^a^	0.57	0.001^b^	Strongly compatible	Harmful
Age	0.51	0.001^b^	Strongly compatible	Harmful
Systolic blood pressure	0.50	0.001^b^	Strongly compatible	Harmful
Physical activity^c^ (MET min/week)	−0.31	0.01^b^	Moderately compatible	Beneficial
Physical activity^d^ (total Kcal)	−0.30	0.01^b^	Moderately compatible	Beneficial
Six-minute walk^e^	−0.29	0.01^b^	Moderately compatible	Beneficial
Sit-to-stand^f^	−0.27	0.02^b^	Weakly compatible	Beneficial
Balance^g^	−0.27	0.01^b^	Weakly compatible	Beneficial
Diastolic blood pressure	0.16	0.13	Unclear	Harmful
Hand Grip Strength^h^	0.02	0.94	Trivial	Don’t use
Body Mass Index	−0.04	0.75	Unclear	Need more data

^a^ The total number of risk factors that the participant had from the following list: Smoking, any previous heart incidents, previous stroke, systolic blood pressure ≥140 mmHg, and whether they were on blood pressure medication.

^b^ Statistically significant.

^c^ A tally of the minutes of physical activity accrued over a typical week, as assessed by the Yale Physical Activity Survey.

^d^ Overall Kcal estimated after applying an intensity code to each of the physical activities documented in the Yale Physical Activity Survey.

^e^ Distance covered in 6 min of continuous walking in a 30-m corridor.

^f^ Number of times a participant can move between sitting on a chair and standing (unaided) in 30 s.

^g^ Assessed by a progressive, 4-stage balance test.

^h^ Combined highest score from each hand achieved from squeezing a hand-grip dynamometer.

Model A of the multivariate regression explained 16% of the variance and was a significant predictor of PWV, *F* (4, 75) = 3.52, *p* < 0.01, with an *R* ^2^ of 0.16. The predicted effect on PWV was: 17.09 + 0.03 (Grip strength) - 0.79 (Balance) −0.09 (Sit-to-Stand) - 0.01 (6-min walk).

Model B explained 50% of the variance and was a significant predictor of PWV, *F* (11, 68) = 6.08, *p* < 0.001), with an *R* ^2^ of 0.5. The predicted model in this regression analysis was: 1.05–0.73 (balance) - 0.09 (Sit-to-stand) - 0.02 (diastolic blood pressure, DBP) + 0.56 (Number of risk factors) +0.09 (age) + 0.06 (systolic blood pressure, SBP) + 0.02 (grip strength) and betas of +0.001 were found with the remaining variables, 6-min walk, total energy expenditure Kcal/week, total PA min/week, and BMI, suggesting small predictive contributions.

Model C explained 45% of the variance and was a significant predictor of PWV, *F* (3, 76) = 20.53, *p* < 0.001, with *R* ^2^ = 0.45. The participant’s predicted PWV using this model is as follows: 3.71 + 0.59 (number of risk factors) + 0.11 (age) + 0.05 (SBP).

## 4 Discussion

The aim of the present study was to explore the relative importance of strength, balance and physical activity on arterial stiffness in an older population. Our key findings indicate risk factors such as older age, higher SBP and a larger number of health risk factors were the strongest predictors of higher PWV. Moreover, higher physical activity levels (measured through self-report physical activity and 6-MWT) are better predictors of PWV than balance and hand-grip strength and sit-to-stand. Our findings are supported in previous research on males older than 50, but slightly younger than our participants. For example, among 54- to 75-year-old men, but with similar blood pressures, endurance-trained men showed significantly attenuated arterial stiffness indexes (25% lower), and Augmentation Index scores (36% lower) than their physically inactive counterparts (Vaitkevicius et al., 1993; Vlachopoulos et al., 2010). Our study suggests that engaging in regular aerobic exercise more strongly correlated with lower arterial stiffness and SBP than resistance training (as measured by hand-grip strength and sit-to-stand ability which are indications of muscular fitness). Nevertheless, a combination of the two types of exercises has been found in other studies to be advantageous to PWV and SBP. A recent study by Tardelli, et al. (2022) on spontaneously hypertensive rats (SHR) showed that after 8-weeks of combined aerobic and resistance training, arterial stiffness was reduced, resulting in a better autonomic balance to the heart and potentially explaining the lowering of blood pressure. SHR are inbred genetic rats with experimental hypertension created to mimic human essential hypertension for research trials (Gardner, et al., 2011)). Moreover, Shimojo et al. (2018) studied menopausal effects in ovariectomised SHR suggesting that combined aerobic and resistance training reduced mean arterial blood pressure by 12% compared to their physically inactive counterparts, heart rate by 8%, vascular sympathetic modulation by 40%, and improved overall baroreflex sensitivity. Perhaps the resistance training types (ladder adapted to rats, *versus* lifting weights or using exercise bands by older adults) played a role in these differences in findings.

The weak (sit-to-stand and balance tests) to moderate (total self-report physical activity and 6-MWT) relationship between these tests and arterial health is possibly due to the nature of the impact of the physical activity on the arterial walls. Tanaka and Safar (2005) have hypothesised that short-term physical activity (measured self-report physical activity volume) likely improves arterial stiffness via improvements in vasoconstrictor tone in smooth muscle tissue as well as enhanced nitric oxide bioavailability which regulates brachial artery elasticity. Conversely, long-term regular physical activity appears to prevent structural changes in the arterial walls that relate to the covalent crosslinking between long-lived proteins (such as collagen) and advanced glycation end-products (Takeuchi, 2020). While aerobic training introduced following the onset of cardiovascular disease can work to reduce arterial stiffness in older adults, the longevity of the improvements is unclear (Madden et al., 2013), which may explain the beneficial, but weak to moderate relationship between physical activity and PWV in our study. Moreover, our results indicated a weak correlation between PWV and the anaerobically-powered sit-to-stand test, whereas there was a moderate correlation between PWV and the aerobically-powered 6-MWT (See Table 3). However, a recent systematic review and meta-analysis demonstrated no significant correlation between PWV and high intensity interval training as well as moderate-intensity interval training (Way et al., 2019).

The strength of the relationship between a higher number of risk factors and increased PWV may help to explain the moderate relationship between overall physical activity and PWV (see Table 3). In this regard, findings by Madden et al. (2013) suggest that there is at least some irreversible arterial damage that occurs in individuals with multiple risk factors for arterial stiffness, such as advanced age, diabetes, hypertension or hypercholesterolemia. Therefore, in those participants with one or more risk factors for cardiovascular disease, the beneficial effect of physical activity on PWV is not strong enough to overcome the damaging effect of the risk factors on the arterial walls (and therefore off-set the beneficial relationship between physical activity and PWV).

Regarding the relative importance of each of the fitness criteria, our findings suggest that overall physical activity volume (self-report calculated to Kcal and MET min/week) and aerobic fitness (6-MWT) (see Table 3) are moderately related to PWV, but grip strength had no effect on PWV and sit-to-stand was only weakly compatible with PWV. These findings showed higher significance in previous meta-analyses by Zhang et al. (2018) as well as Ashor et al. (2014), indicating improvements in PWV are associated with aerobic exercise, and trended towards significance with combined aerobic and resistance training. These changes may be due to improvements in peak oxygen uptake (Deiseroth et al., 2019) and endothelial function (Zhang et al., 2018). Unlike the intervention studies reported in the meta-analyses above, our cross-sectional study provides evidence supporting the role of habitual daily physical activity in supporting arterial health in older people.

Finally, our study indicates that BMI is a poor predictor for arterial stiffness (see Table 3). While there seems to be a paradox regarding the arterio-protective nature of metabolically-healthy obesity on arterial stiffness (Yuan et al., 2020), particularly in the young-middle aged populations (Corden et al., 2013; Yuan et al., 2020), the absence of a relationship between BMI and arterial stiffness in our older cohort is likely due to the BMI measurement ( $\frac{Weight\;\left(kg\right)}{{\left(height\left(m\right)\right)}^{2}}$ ) itself. As height is static, changes in BMI would be due to the participant’s weight, which is influenced by changes in either the fat mass, or lean mass of the individual. An increase in fat mass is likely to be associated with an increase in arterial stiffness in middle-age to older populations (Benetos et al., 2009; Corden et al., 2013), while an increase in lean leg mass is associated with reduced arterial stiffness (Snijder et al., 2004). Therefore, an increase in BMI could either be beneficial or not depending on the reason for its elevation. Considering that BMI rises with increased fat mass or reduced muscle mass, it would be prudent to investigate age-related changes, such as sarcopenia. Low thigh cross sectional area (Huisman et al., 2015), and sarcopenia (Ochi et al., 2010) in older men have also been associated with increased arterial stiffness. Therefore, as BMI is unable to differentiate between fat and lean mass, it is perhaps not surprising that it was not associated with arterial stiffness in our study. Future research should measure body composition rather than BMI to elucidate the relationship between fat mass, muscle mass and PWV.

There are a number of limitations that need to be addressed in our study. As participants opted into the study, there may be bias towards more physically fit individuals and there were more women in the study. Each of the three villages recruited into the study had different participant profiles (See Table 2). However, as this was not an intervention study, and all data was pooled prior to analysis, the diversity of the participants in each of the retirement villages likely improved the ecological validity of our findings to the general population. This study took place over a year, therefore, participants were tested in different seasons. This seasonal change may have impacted participant’s levels of physical activity and that could have impacted arterial stiffness. The small sample size of this study limits the extent to which the findings can be applied to the older population in general and there were predominantly women. Some participants had CVDs and others did not. We tried to accounted for these individual health variations by creating the “number of (CVD) risk factors” variable (smoker, heart incidents, previous stroke, systolic blood pressure ≥140 mmHg, and/or taking anti-hypertension medication). We also collected a list of all the medications participants were taking. We controlled for participants taking anti-hypertension-specific medications (namely, ACE Inhibitors, ARBs and Beta-Blockers) as they directly impact PWV and they were among the most commonly taken in our sample. Stratifying the study population based on medication use, particularly corticosteroids and other relevant drug groups, allows for a more granular examination of their impact on arterial stiffness. The scope of this study, however, did not allow us to rationalise controlling for other medications as they were not frequent enough among participants, the could have complex interactions our study would not be able to detect, and the combinations varied too much from person to person. Body composition (BIA or DEXA) was not collected, nor was leg strength, which could have been informative variables to assess. Some participants had a light meal, i.e., were not fasted, prior to measurement due to medication needing to be taken with food. Although the recommendation is to have at least two measurements taken of cfPWV with a difference <0.5 m/s (Townsend et al., 2015), several PWV measures in our study took up to 50 min to get a quality-controlled tick, so in some instances, we were only able to collect one PWV measure. With participants scheduled back to back, we did the best we could to fit in two PWV measurements but in several cases, we had to settle for one due to the next participant arriving and limitations on room bookings.

Nevertheless, some of the limitations provide insight for future studies. For example, when measuring PWV using tonometry in older adults who may have larger necks and less elastic skin, it is important to allow for an additional 30 min to increase the likelihood of obtaining a second PWV measure. Also, in the absence of muscle mass bioimpedance, future studies might include measuring calf circumference in addition to grip strength for a muscle mass estimate for older adults (Quiñonez-Olivas, et al., 2016). It could have also been interesting to investigate the number of years since hypertension diagnosis.

Given the prevalence of comorbidities and polypharmacy (multiple medications taken simultaneously) in older adults, investigating potential synergistic or antagonistic effects between different drug classes is important to understand their broader interactions (Sinnott and Bradley, 2015). The inclusion criteria of designing such a study would need to control for the specific mix of drug classes taken, which was another limitation of this study. For example, the presence of diabetes for a long time is correlated with accelerated arterial stiffening indirectly through multiple mechanisms including, inflammation, endothelial dysfunction, elevated blood pressure, and changes in the structural components of the arterial walls (Petrie et al., 2018). By mitigating inflammation, corticosteroids might theoretically contribute to improved vascular health, making arterial stiffness a co-beneficiary of anti-diabetes medication (Laurent et al., 2006; Lunder et al., 2021). Polypharmacy can complicate the interpretation of associations, so careful consideration of medication interactions is warranted, particularly in the development of novel treatments (Salwe et al., 2016). Although it was beyond the scope of this study, future research should aim to delineate the specific mechanisms through which medications impact PWV and explore optimal therapeutic strategies that balance the benefits and potential risks associated with different drug classes (Janić et al., 2014).

In conclusion, this study reinforces the importance of habitual, lifelong physical activity in maintaining arterial health in an older population. However, the adverse effect of high systolic blood pressure, increasing age and overall numbers of cardiovascular risk factors had a stronger ability to predict arterial stiffness in an older population than fitness-based tests. Therefore, while engaging in physical activity later in life will have a beneficial effect on vascular health, regular physical activity throughout the lifespan, amongst other cardiovascular risk-reduction strategies, will have larger impact on pulse wave velocity in aging adults.

## Data Availability

The raw data supporting the conclusions of this article will be made available by the authors, without undue reservation.
